# Multiple Glioblastomas Ablation by Laser Interstitial Thermal Therapy (LITT): A Rare Case

**DOI:** 10.7759/cureus.66726

**Published:** 2024-08-12

**Authors:** Ahmed Abdulsalam Ali Bakrbaldawi, Umar Al-Sheikh, Hongjie Jiang, Junming Zhu

**Affiliations:** 1 Neurosurgery, The Second Affiliated Hospital of Zhejiang University, School of Medicine, Hangzhou, CHN; 2 Clinical Research Center, Neurological Diseases of Zhejiang Province, Hangzhou, CHN; 3 Epilepsy Center, The Second Affiliated Hospital of Zhejiang University, School of Medicine, Hangzhou, CHN; 4 Neurosurgery, The First Affiliated Hospital of Zhejiang University, School of Medicine, Hangzhou, CHN; 5 Neurobiology and Neurology, The Fourth Affiliated Hospital of Zhejiang University, School of Medicine, Yiwu, CHN; 6 NHC and CAMS Key Laboratory of Medical Neurobiology, MOE Frontier Science Center for Brain Research and Brain-Machine Integration, School of Brain Science and Brain Medicine, Zhejiang University, Hangzhou, CHN

**Keywords:** vasogenic edema, blood-brain barrier, brain tumors, litt, multiple glioblastomas

## Abstract

Multiple glioblastomas (GBMs) are aggressive, malignant, and sporadic brain tumors. We present the case of a 58-year-old patient with two GBMs in the right frontal lobe and associated edema. The patient presented with sudden left limb weakness accompanied by abnormal gait for five consecutive days. Magnetic resonance-guided laser interstitial thermal therapy (MRg-LITT), a minimally invasive technique that disperses thermal energy was used to cauterize the deep-seated brain lesions. Following two sessions of MRg-LITT, the patient showed full remission from symptoms. However, the disruption of the blood-brain barrier (BBB) induced vasogenic edema surrounding the necrotic GBMs. Post-operative nine-month MRI images revealed severe vasogenic edema and compression on the ventricles, shifting the midline toward the left side. Therefore the patient underwent an emergency craniectomy and continues to live with close follow-ups. Here, we established that LITT procedures were effective in cauterizing GBMs with no recurrence.

## Introduction

Glioblastomas (GBMs) are highly malignant brain tumors that emanate from glial cells [1]. These intracranial neoplasms are associated with poor prognosis due to rapid growth, invasiveness, and high recurrence rates [2]. Multiple GBMs are two or more lesions, at least 1 cm apart in the same or different brain lobes, categorized as multicentric or multifocal based on their dissemination [3]. Multicentric GBMs are separate synchronous tumors that lack apparent neural or vascular connections, unlike multifocal lesions where clear connections can be observed [3]. Laser interstitial thermal therapy (LITT) is a minimally invasive technique to cauterize intracranial lesions guided by real-time magnetic resonance [4,5]. The magnetic resonance-guided laser interstitial thermal therapy (MRg-LITT) technique enables the treatment of deep-seated lesions with minimal complications, often unsuitable for surgical resection [6]. In addition, LITT can disrupt the blood-brain barrier (BBB), promoting the feasibility of adjuvant pharmacotherapy and thus offering a safer approach for patients with multiple GBMs [4,7].

## Case presentation

A 58-year-old male patient presented at the emergency with sudden left limb weakness accompanied by abnormal gait for five consecutive days. He denied any history of vomiting, headache, or dizziness. His past medical history included hypertension, which he managed using an anti-hypertensive drug (Norvasc®) once daily. He further denied any other past medical or surgical history with no significant personal or family-related diseases. In addition, the patient admitted to having a stressful and sedentary lifestyle, often with alcohol and cigarette abuse. The preliminary blood test revealed a positive hepatitis B surface antibody (HBsAb) and hepatitis B core antibody (HBcAb), but a negative hepatitis B surface antigen (HBsAg), indicating that the patient recovered from an acute hepatitis B infection. The patient further stated about not being aware of the hepatitis B infection and did not receive any treatment or vaccine except for the SARS-CoV-2 virus (COVID-19).

**Figure 1:**
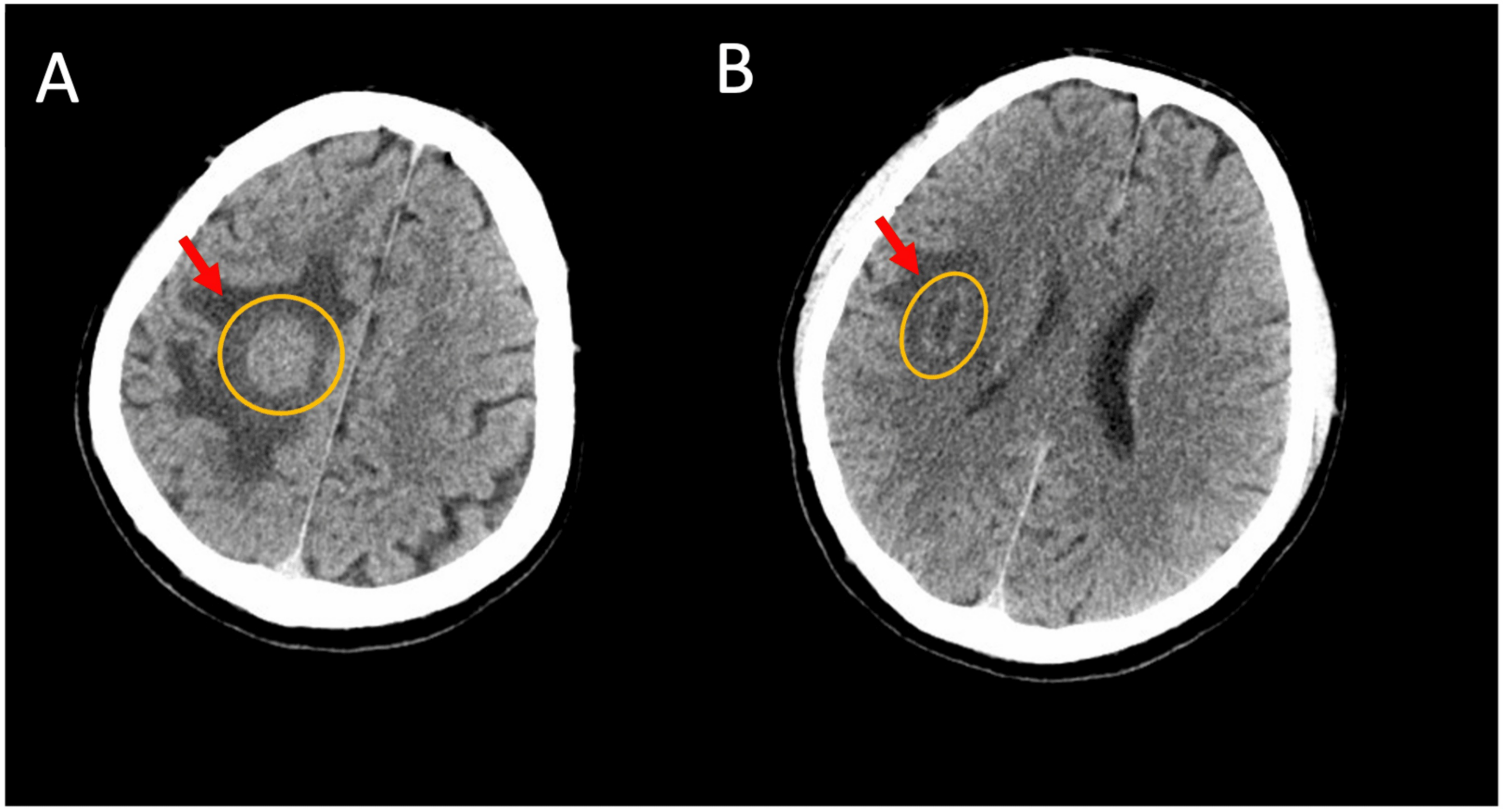
Axial CT scan (A) Right frontal lobe glioblastoma 3.5 cm × 3.1 cm (orange) surrounded by edema (red arrow); (B) 2.5 cm × 1.1 cm glioblastoma (orange) and edema (red arrow)

**Figure 2:**
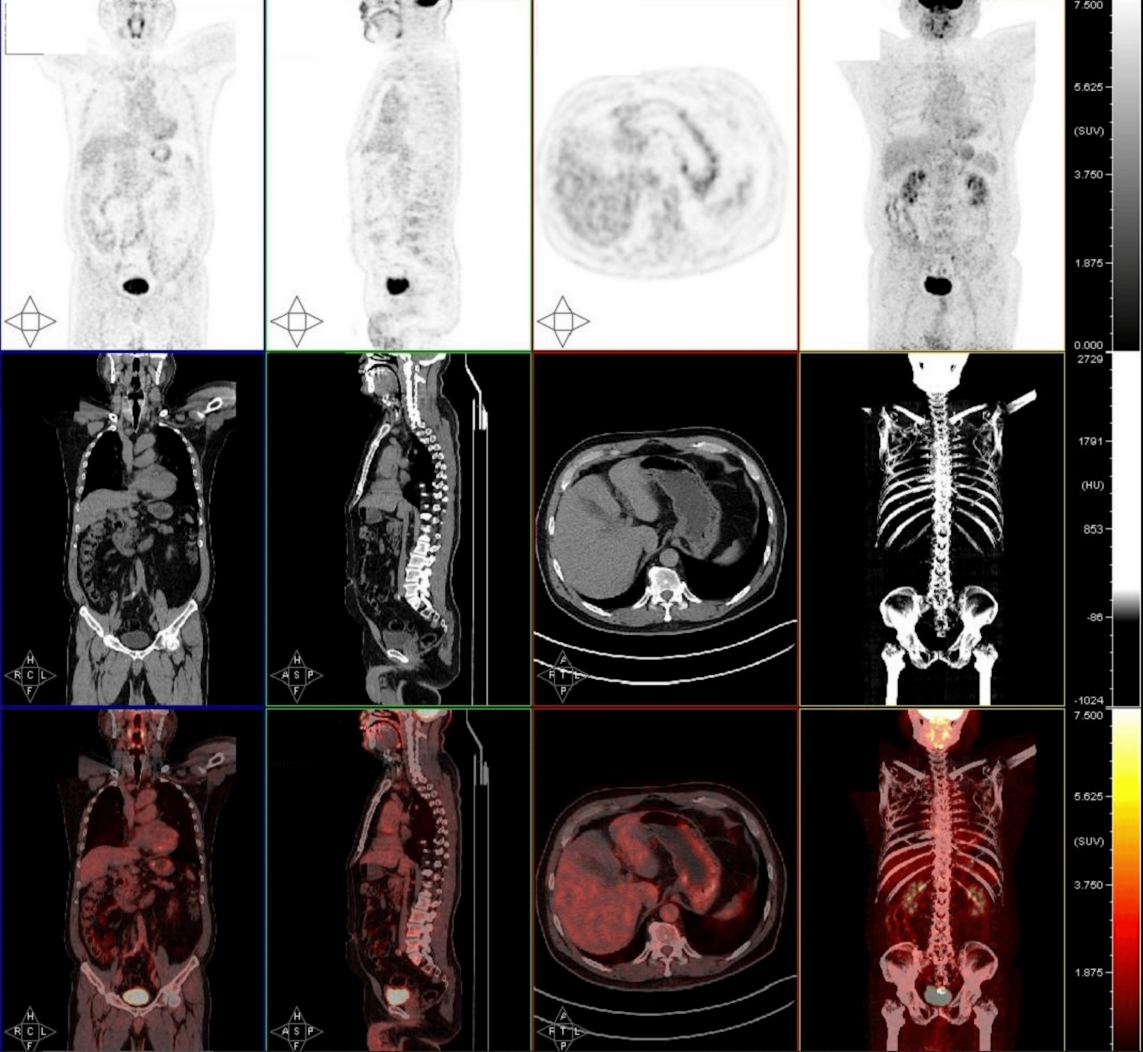
Whole-body PET scan No metastatic tumors detected

An emergency computed tomography (CT) scan of the head was performed, which revealed two abnormal masses in the right frontal lobe, measuring 3.5 cm × 3.1 cm and 2.5 cm × 1.1 cm with no neural or vascular connections, and accompanied by surrounding edema (Figure 1). A whole-body positron emission tomography (PET) scan was performed which did not reveal any metastatic lesion (Figure 2). Several treatment approaches were discussed with the patient and family members. They eventually opted for the LITT minimally invasive procedure as the preferred treatment modality. Two sessions of MRg-LITT were scheduled with an interval time of two months for each brain lesion to minimize post-operative edema. Written informed consent was obtained from the patient to be included in this study for publication.

MRg-LITT procedure

**Figure 3:**
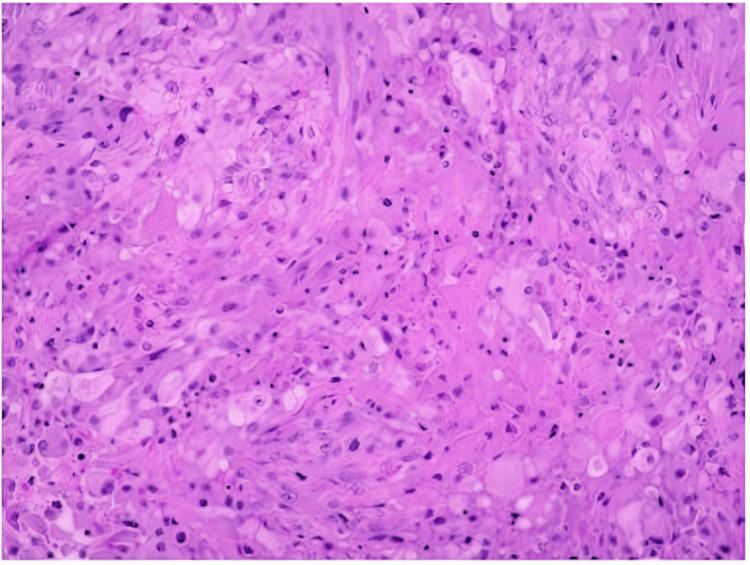
Biopsy of tumor obtained before the ablation procedure Presence of malignant neoplasm with epithelioid cytomorphology, diffused distribution pattern with cell necrosis. Glioblastoma, IDH wild-type, WHO grade 4. Malignant tumor with epithelioid cells, diffuse distribution, GFAP (+) (individual cells), Olig-2 (-), Ki-67 60%, P53 70%, IDH1 (H09) (-), NeuN (-), NF (-), ATRX (+), Syn (-), CD34 (+), EMA (+), H3K27M (-), H3K27Me3 (+), CK (AE1/AE3) (-), CD31 (partial) (+), S-100 (individual cells) (+), SOX10 (-), CgA (-), BRAF-V600E (-), BRAF-PC (+), INI-1 (FlE), Desmin (-), CAM5.2 (-), SALL4 (-), IDH1 gene mutation (wild type), IDH2 gene mutation (wild type), BRAF gene V600E mutation (negative), TERT gene promoter mutation (mutant - C250T), CDKN2A/CEN7 FISH (consistent with CDKN2A gene deletion) with absent glomerular vascular proliferation

The probe trajectory was determined pre-operative to ensure the safety of critical structures using the Sinovation® surgical planning system (Beijing, China). A stereotactic CT with contrast was performed on the morning of surgery to visualize the lesion appropriately. The first MRg-LITT procedure was performed for the largest (3.5 cm × 3.1 cm) tumor. The patient was placed in a supine position under general anesthesia and the entry site was established with a Leksell stereotactic frame. A burr hole was prepped using a battery-powered hand drill and the dura mater was cauterized using unipolar electrocoagulation. A stereotactic biopsy was then performed to extract a portion of the tumor tissue for pathological analysis (Figure 3). Subsequently, a thin optic fiber catheter with an outer diameter of 1.55 mm was carefully inserted and connected to the LITT apparatus - LaserRO™ (Jialiang Medical, Hangzhou, China), which includes normal saline channels for cooling. Real-time MRI temperatures were measured during laser ablation.

**Figure 4:**
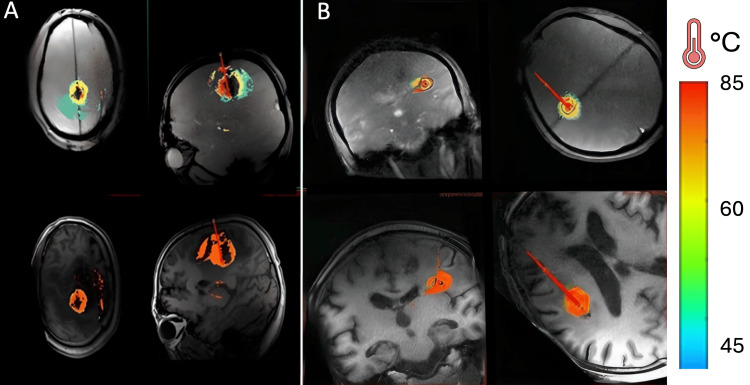
Real-time intraoperative MRI imaging (A) and (B) thermal damage threshold (TDT) of the large and small tumors, respectively, and temperature bar on the left side

**Figure 5:**
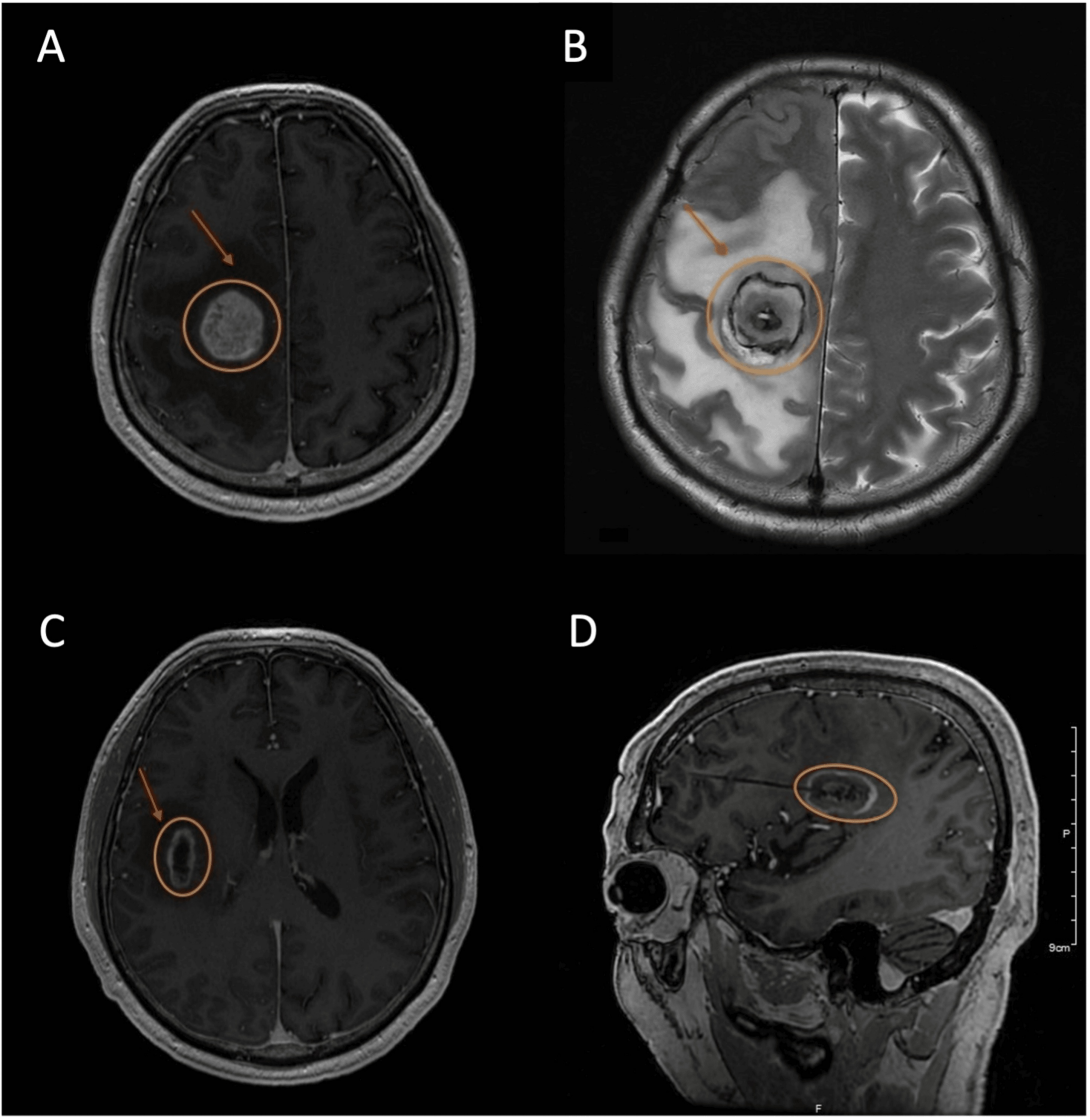
MRI scans pre- and post-operative (A) pre-operative axial T1 FSPGR + contrast MRI; (B) axial T2 FSE ACS MRI 24 hours post-operative of the right frontal lobe large GBM (orange), surrounded by edema (red arrow); (C) pre-operative axial T1 FSPGR + contrast MRI; (D) sagittal T1 + contrast MRI 24 hours post-operative of the smaller tumor necrosis (orange) and edema (red arrow)

**Figure 6:**
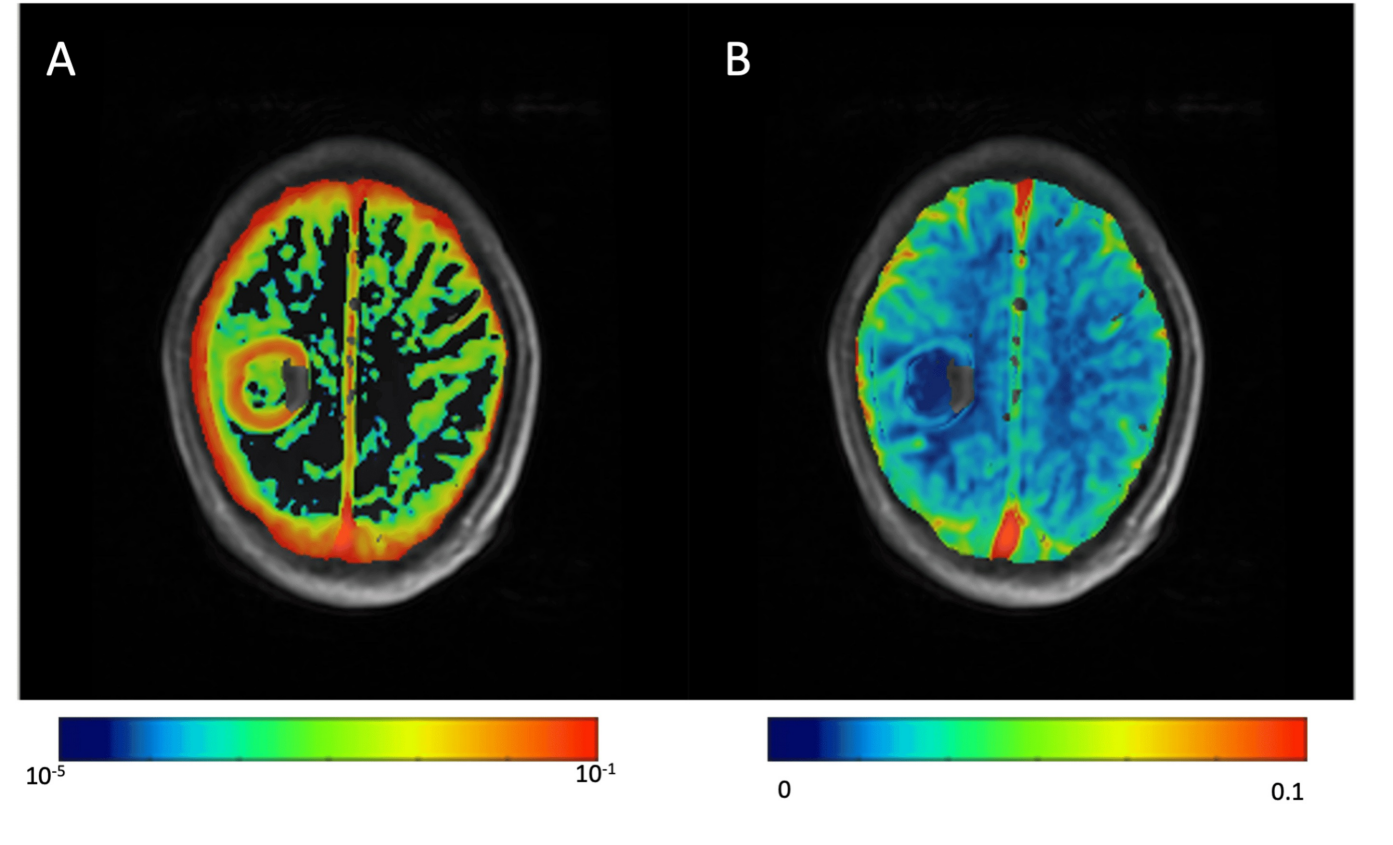
Gadolinium-based contrast DCE-MRI vascular permeability maps (A) An increase in blood-brain barrier leakage rate (Ktrans); (B) blood plasma volume (Vp) in the peri-ablated area was low or absent DCE-MRI: dynamic contrast-enhanced magnetic resonance imaging

A 1064 nm laser was selected due to its power in cauterizing a wider-range area to target the large tumor at 4 W for 54 seconds (s) as a trial for visual presentation of temperature changes, followed by, 15 W for 48 s, 15 W for 300 s, and 15 W for 300 s. The thermal damage threshold (TDT) indicated the region of tissue cauterization (Figure 4). The ablation range was approximately 3.5 cm in length and 2.7 cm in width. A post-operative MRI scan further confirmed the ablation range without complications (Figure 5). Dynamic contrast-enhanced magnetic resonance Imaging (DCE-MRI) corroborated the disruption of the BBB (Figure 6). Similarly, a second procedure was performed for the smaller (2.5 cm × 1.1 cm) tumor after two months using a 980 nm laser at 4 W for 23 s as trial, 12 W for 128 s, 12 W for 150 s, and 12 W for 90 s. A thermal range of 3.1 cm in length and 1.4 cm in width cauterized the tumor. Finally, the optic fiber catheter was immediately removed after each procedure, and the puncture site was sutured and closed appropriately. Dexamethasone sodium phosphate injection 5 mg/mL was administered pre-operative and post-operative.

Post-operative

Following the first procedure, the patient was admitted to the hospital for 14 days with careful follow-ups and physiotherapy. The patient attended chemotherapy sessions pre- and post-ablation as part of the treatment course. Furthermore, the patient presented symptoms of restlessness, refused mental health assistance and eventually he was discharged to return to his hometown. Follow-ups were made by phone every week and no abnormal behaviors/symptoms were reported. Prior to the second LITT procedure, routine blood tests did not reveal any significant concerns. The patient stayed 35 days in the hospital after the second procedure mainly for extended rehabilitation. Muscle weaknesses significantly improved, from grade 1 to grade 4 on the muscle strength scale, after four months following the first LITT procedure.

**Figure 7:**
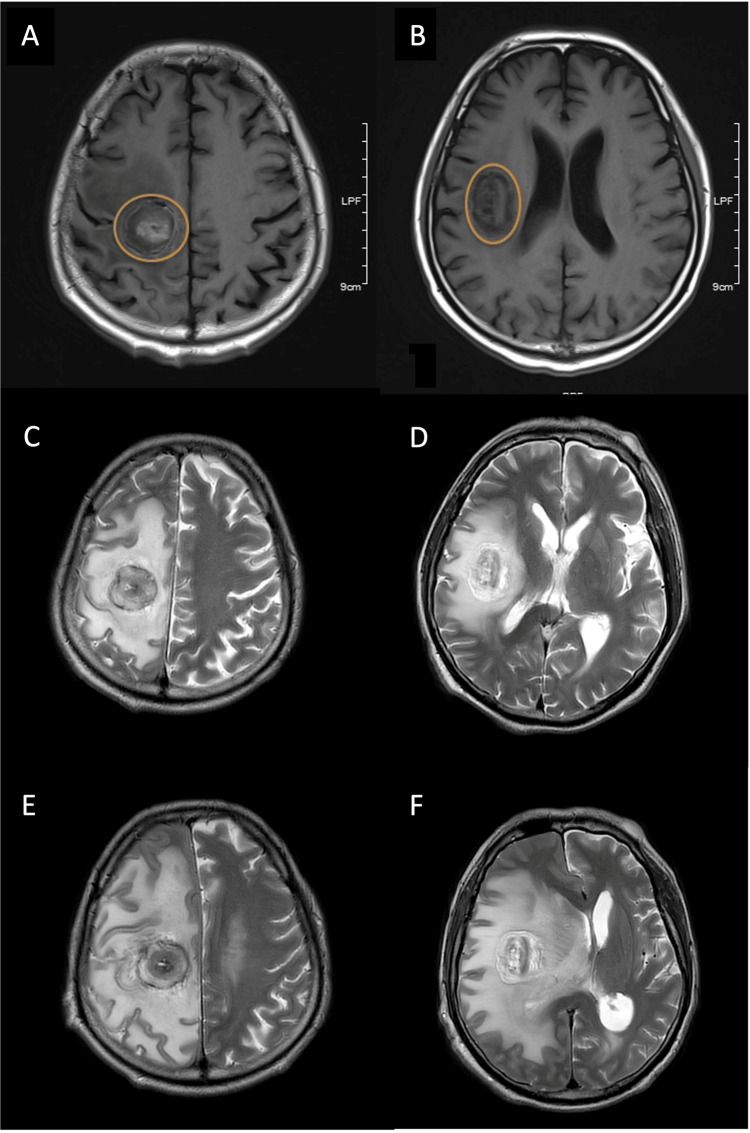
MRI scans at different time points (A, B) Axial T1 MRI of right frontal lobe glioblastomas (orange) three months post-operative; (C, D) axial T2 MRI tumor necrosis six months post-operative; (E, F) axial T2 MRI tumor shrinkage necrosis and vasogenic edema nine months post-operation

By the sixth month, MRI images revealed post-operative vasogenic edema surrounding the necrotic GBMs (Figure 7). In addition, no recurrence of the tumors was observed. The patient was prescribed mannitol, albumin, diuretics, and corticosteroids to manage the vasogenic edema. Unexpectedly, the patient seemed to be unresponsive to these treatments and the family members stated that the patient was not taking the medications accordingly. However, the patient denied the statement of his family members. By the ninth month post-operative, the patient was admitted to the emergency due to acute headache, dizziness, and muscle weakness. MRI scans revealed severe vasogenic edema shifting the midline to the left side and compressing the ventricles (Figure 7). The patient underwent an emergency craniectomy to alleviate intracranial pressures and was responsive to glucocorticoids. No recurrence of the GBMs has been observed 13 months since the first procedure. The patient is currently under close follow-ups with rehabilitation and continues to live without any major issues.

## Discussion

GBMs are classified as either multifocal or multicentric brain lesions. Multifocal gliomas arise from the dissemination or growth of tumor cells through pre-existing pathways such as commissural fibers, cerebrospinal fluid pathways, or local metastasis [3]. In contrast, multicentric gliomas are spatially separated into different lobes or hemispheres. Multiple GBMs tend to exhibit more aggressive behavior compared to solitary GBMs [8]. Multiple lesions are typically detected through CT/MRI scans, and symptoms are often attributable to the size or dysfunction of the affected brain region [9]. However, making a definitive diagnosis solely based on imaging can be challenging.

Standard radiological differentials include cerebral abscess, metastasis, lymphomas, and demyelinating diseases like multiple sclerosis [10]. Despite the multiple treatment options available, multiple GBMs still have poor prognoses due to their aggressive nature and high recurrence rates [11]. There are no standardized treatment guidelines for multiple GBMs, necessitating further investigation and evaluation to better understand this rare neoplasm. To our knowledge, there are few studies that have investigated the surgical outcome of multiple GBMs due to their rarity or misdiagnosis [9,10]. Here, we presented clinical evidence, radiological findings, histopathological characteristics, and treatment strategies for multiple GBMs at our hospital. We mainly focused on LITT as a prominent therapy for multiple GBMs.

The LITT technique offers multiple advantages compared to repeated surgical interventions, including decreased post-operative complications related to wound healing and cerebrospinal fluid build-up [12]. In addition, the LITT technique facilitates a shorter recovery period and safe treatment of deep-seated lesions that would otherwise be unsuitable for surgical resection [6]. This technique also allows for the disruption of the BBB, enabling the administration of adjuvant therapy to enhance the treatment outcome. However, the management of vasogenic edema post-operative still needs further research and appropriate treatments [12] as we observed in this case.

Multiple research studies have examined the use of LITT in managing brain lesions, providing evidence of its feasibility and potential complications [13]. However, further investigations involving larger patient cohorts are necessary to ascertain the long-term clinical outcomes associated with this treatment modality. Although there is a limited understanding of how LITT transiently affects the BBB or blood tumor barrier (BTB), only a few small-scale series have described the spatiotemporal relationship between LITT and the disruption of the BBB/BTB [5,7,14]. In addition, the mechanisms underlying this phenomenon are still in the early stages of comprehension.

Future research directions could explore the correlation between thermal dosage and the spatiotemporal relationship of molecule diffusion across the BBB/BTB, considering the varying sizes of these molecules. A deeper understanding of this aspect could also enhance the sensitivity and specificity of LITT, thereby optimizing its effectiveness and minimizing complications when targeting specific tissues of interest. This further includes the duration of BBB/BTB opening after LITT procedures and treatment with adjuvant therapies. Large-scale studies are required to assess complications when the BBB/BTB is open for a longer duration post-LITT, including external factors such as infection. Moreover, advancements such as faster refresh times for MRI thermography and improved temperature resolution could contribute to refining the ablation process, particularly near vital structures.

## Conclusions

We successfully used LITT as a prominent surgical option to treat multiple GBMs in one patient. Follow-up neuroimaging revealed tumor shrinkage and the patient remained free of any post-operative complications several months after laser ablation. In addition, we observed significant improvements in symptomatology without recurrence of the tumors over the course of a year. However, it is also important to mention vasogenic edema which occurred after the disruption of the BBB. The patient was careless about the severity of such disruption by ignoring guidelines and medications. Thus, the patient required an emergency craniectomy to relieve intracranial pressures. In short, LITT offers a minimally invasive approach to the treatment of deep-seated or difficult-to-access brain lesions while minimizing neurological deficits. However, further follow-ups and prospective studies are required to evaluate this promising intervention for multiple GBMs.
